# Implementing a teleophthalmology referral platform in routine practice: Understanding a digital health intervention implementation using normalisation process theory

**DOI:** 10.1177/20552076241303812

**Published:** 2025-01-31

**Authors:** Sarah Abdi, Dilisha Patel, Josie Carmichael, Konstantinos Balaskas, Ann Blandford

**Affiliations:** 1 UCL Interaction Centre, 4919 University College London, London, UK; 2 Global Disability Innovation Hub, 4919 University College London, London, UK; 3 4960 Moorfields Ophthalmic Reading Centre & Clinical AI Lab, Moorfields Eye Hospital, London, UK; 4 59899 UCL Institute of Ophthalmology, 4919 University College London, London, UK

**Keywords:** **Keywords**Teleophthalmology, normalisation process theory, community optometrists, opthalmologists, primary eye care, clinical referral

## Abstract

**Objective:**

Digital health interventions have the potential to improve clinical processes and patient outcomes; however, many face challenges during the adoption and implementation stages, hindering their overall impact. Our study uses normalisation process theory (NPT) as a theoretical approach to explore the facilitators and barriers to the implementation of a teleophthalmology referral platform in the United Kingdom, as an illustrative case of the implementation of a digital health intervention in routine practice.

**Methods:**

Semistructured interviews were conducted with 24 health professionals (18 optometrists and 6 ophthalmologists) involved in the implementation of a teleophthalmology referral platform. NPT guided data collection and analysis.

**Results:**

Most participants were ready to engage with the teleophthalmology referral platform, recognising its potential value and benefits. However, during implementation, participants’ perceptions varied; a major factor was whether their expectations from the technology were met, particularly regarding the feedback from the secondary eye care component of the referral platform. Several additional factors were identified that would influence the adoption of the platform. These included individual aspects (e.g. participants’ IT skills), technology-related factors (e.g. the time required to complete referrals) and organisational factors (e.g. investment in community optometry services).

**Conclusions:**

To successfully implement the teleophthalmology platform into routine practice, particularly on a large scale, multiple factors at different levels must be considered. This study highlights the complexity associated with implementing digital health interventions in routine practice and the contribution of NPT in untangling some of these complexities.

## Introduction

The potential of digital health to improve the efficiency of healthcare processes, enhance patients’ outcomes and reduce pressures on the healthcare workforce has been widely recognised, particularly following the COVID-19 pandemic.^1,2^ There have been successes in the deployment of digital health innovations in routine healthcare practice over the past few years; for example, ophthalmology had witnessed an acceleration in the adoption of synchronous (live review) teleconsultations.^3,4^ Asynchronous teleophthalmology, where the review and consultation occur at a later time, has also been deployed in the UK healthcare system and in other regions globally.^4,5^ Similar trends have been observed in the accelerated adoption of digital health innovations in various areas that support the delivery of clinical care and self-management of patients.^6,7^ Despite these trends, digital health innovations have not realised their potential, and many innovations encounter difficulties during the implementation and deployment stages.^4,8^ Many of the common adoption barriers are arguably related to the complex nature of the healthcare system that digital technologies aim to support as well as to the digital technology itself.^8–11^ Healthcare systems are complex due to the interdependencies between their agents, which interact with each other producing actions that are uncertain and unpredictable.^10^ In basic terms, if these systems were given the same input (a patient with a healthcare requirement), they may produce different outcomes (variable quality of the care provided and clinical outcomes). The complexity related to the digital health intervention can arise because of the multiple components involved, multiple stakeholder groups and behaviours targeted by the intervention.^12^ These complexities need to be considered when designing, developing, evaluating and implementing digital interventions within the healthcare system to avoid potential failures due to oversimplification. The Medical Research Council updated framework to develop and evaluate complex interventions in healthcare recommends various strategies to account for these complexities.^13^ Engaging with stakeholders at various stages of research is listed as a core element for any complex intervention research as this contributes to a better understanding of what would or would not work in real-world practice, extending the focus of intervention research from solely addressing effectiveness or efficiency questions.^13^ The guidelines also emphasise the importance of adopting a theory-based approach in developing and/or evaluating complex interventions. This could help in examining the intervention mechanisms of action as well as how they interact with the context and influence one another. This knowledge may contribute to better understanding how an intervention can achieve its desired outcomes and aid in refining the intervention, ultimately facilitating the integration of technology into routine care.

In this article, we report the findings of a theory-based evaluation of the use of a teleophthalmology platform in routine practice by community optometrists and ophthalmologists. This serves as an example of theory-based evaluations of digital health interventions. It also highlights both the benefits and challenges of using this type of approach as well as the potential and challenges of implementing digital health interventions in routine practice.

### The digital health intervention and the problem it addresses

Retinal conditions like age-related macular conditions (AMD) and diabetic retinopathy are among the most common eye conditions and leading causes of blindness in people aged 50 years and older.^14^ Identifying these conditions accurately and referring patients with suspected cases to secondary care in a timely way is crucial to avoid preventable sight loss. This not only improves individuals’ quality of life but also benefits the healthcare system by reducing the economic burden caused by blindness and vision loss.^15^ However, the reality is that ophthalmology clinics in hospitals are overstretched, leading to delayed appointments and potentially negative impacts on patients. Many factors are contributing to an increased burden on secondary eye care, including an increase in the number of referrals from community optometrists that require review and a reduced number of ophthalmologists per capita.^16,17^ There is an urgent need for innovative solutions to reduce the burden on secondary eye care, address the challenges with referrals, and improve patients’ access to timely care. Digital health interventions, alongside interventions like improving optometrists’ skills and knowledge and referral refinement schemes, are among the proposed solutions to improve the accuracy of referrals and reduce unnecessary visits to secondary eye care.^18^

**Figure 1.**
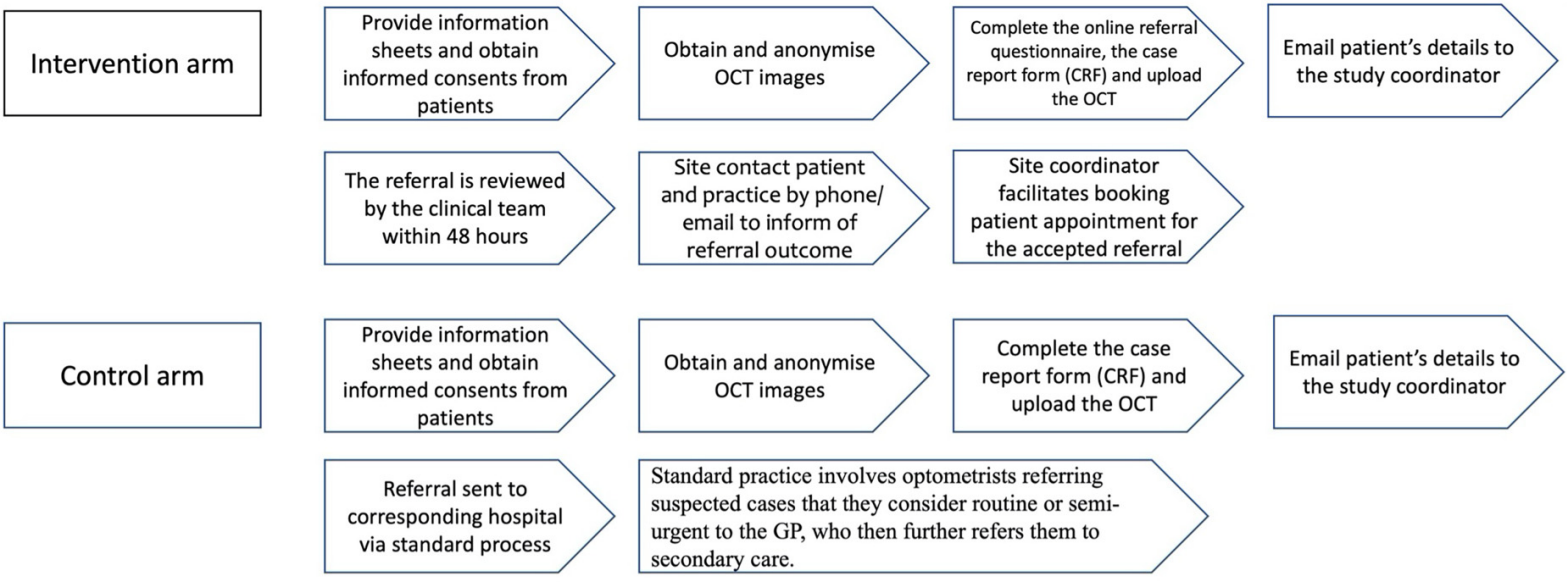
Pathways followed by the intervention and control arms in the HERMES trial.

Teleophthalmology is one of the core digital technologies that hold promise to improve the quality of care in ophthalmology.^4^ The COVID-19 pandemic reignited interest in the potential of teleophthalmology which had previously undergone trials with limited success in clinical practice.^4^ Referrals via teleophthalmology technology are typically initiated by primary care professionals such as community optometrists.^19^ These referrals involve attaching images such as optical coherence tomography (OCT) and/or fundus images and are usually reviewed by ophthalmologists in a hospital setting.^19^ The potential of these technologies to improve the efficiency of the referral process and enhance patient satisfaction by reducing unnecessary visits to secondary care has been well-documented.^20–22^ In particular, asynchronous teleophthalmology is believed to offer advantages over synchronous methods because it provides healthcare providers with the flexibility to review referrals at a later time, which can be more cost-effective.^4^ However, most of the evidence regarding teleophthalmology within the context of retinal disease referrals comes from effectiveness or efficiency trials or studies, focusing on its potential to reduce the number of referrals that require review at secondary care.^4,5,18^ Limited knowledge exists on the factors affecting the efficacy of these interventions and/or integration into practice. This information is needed for the adoption and long-term sustainability of teleophthalmology platforms for referrals. This study aimed to investigate community optometrists’ and ophthalmologists’ acceptance of, and barriers and enablers for, the adoption of a teleophthalmology platform supporting referral pathways of retinal conditions between community optometrists and secondary eye care. The study was conducted in parallel with the ‘Teleophthalmology-enabled and AI-ready referral pathway for community optometrists’ referrals of retinal disease trial’ (the HERMES trial), which is an interventional superiority cluster randomised trial that aims to compare standard practice for referral of suspected retinal diseases with a teleophthalmology digital link between community optometrists and hospital eye services.^23^ The differences between the pathways followed by practices in the intervention and control arms are presented in Figure 1. The practices in the control arm follow the routine practice of referring patients to secondary eye care.

### Theoretical approach

Normalisation process theory (NPT) was used as the theoretical lens that guided the data collection and analysis of this study.^24^ The detailed justification for using NPT as the theoretical foundation for this study has been provided elsewhere.^25^ In brief, NPT was originally developed to explain the implementation processes of complex healthcare interventions.^24^ Some of the key questions that NPT addresses include what types of work individuals and groups undertake to facilitate implementation, how they carry out that work and what impact it has.^26,27^ Normalisation in NPT is defined as integrating the complex intervention into routine care, where the intervention becomes part of everyday practice.^26^ The theory has four core constructs:

1. coherence (making sense of the intervention);
2. cognitive participation (preparedness to engage with the intervention);
3. collective action (work done to make a complex intervention workable) and
4. reflective monitoring (appraisal of the intervention).^26,27^

These constructs have remained stable across contexts and have traditionally been used to understand facilitators and barriers to the implementation of complex health interventions, including digital technologies, in routine practice.^26,28–30^ NPT was chosen as the theoretical framework for this study as our aim was to understand what would facilitate or hinder the implementation and integration of a teleophthalmology referral platform in routine practices of community optometrists and ophthalmologists. This theoretical approach complements recently published work that conducted a thematic analysis of the experiences of patients and health professionals using the teleophthalmology platform for the referrals of suspected retinal conditions.^31^

## Methods

The protocol for this study has been published elsewhere.^25^ The protocol provides a detailed description of the methods used to explore the views of patients and healthcare professionals on the use of innovative technologies in the referral pathways between community optometry practices and hospital eye services in England. This article focuses on the experiences of healthcare professionals (community optometrists and ophthalmologists), with most participants being users of the HERMES teleophthalmology platform. Methods to identify and recruit healthcare professionals in this study are briefly described below.

### Participant identification

Community optometrists recruited to this study were participating in the HERMES trial. Optometrists were recruited from two groups of practices: those using the HERMES teleophthalmology platform to refer patients with suspected retinal conditions to hospital eye services (HERMES intervention arm), and practices following their usual practice to refer suspected retinal cases (HERMES control arm). No restriction was applied on the level of experience community optometrists had, as it was important to include views from optometrists with various levels of experience. Ophthalmologists recruited to the study were all triaging referrals received from community optometrists via the HERMES teleophthalmology platform. They were local principal investigators (PIs) of the HERMES trial at four sites or clinical fellows designated by the local PIs to triage referrals at those sites. Ophthalmologists had a minimum of 2 years’ experience of independent practice in retinal clinics in hospital eye services.

### Methods of approach

Sixteen community optometry practice managers (10 from the intervention arm and 6 from the control arm) were invited to participate in the study, and the invitation was extended to other optometrists within their practices. Additionally, seven ophthalmologists (4 PIs and 3 clinical fellows) were invited to participate in the study. The potential participants were invited via email which included details about the aim of the study, what their participation would entail, the participant information sheet and a copy of the informed consent form. Nonresponders were followed up with an email and/or a phone call to answer any questions they had about the study.

### Recruited participants

A total of 24 health professionals were recruited to the study: 18 optometrists and 6 ophthalmologists. The 18 optometrists were recruited from 14 practices: 9 from the intervention arm and 5 from the control arm. This group included 11 practice managers or lead optometrists and 7 optometrists identified by their managers. The six ophthalmologists were three principal investigators and three clinical fellows, recruited from four hospitals. One PI ophthalmologist did not have time to participate; no participants dropped out.

### Data collection

The interviews took place between December 2021 and May 2022. All interviews were conducted by SA, who had a PhD that included training in digital health and qualitative methods. Although she was employed on the HERMES project, she had not developed a personal relationship with any participants prior to the study. Participants were informed that she did not have a background in optometry or ophthalmology; this enabled her to probe participants’ experiences without making assumptions about their clinical practice.

Most of the optometrist interviews (n = 14) were conducted via videoconference, and the remainder were conducted via telephone (n = 4). Four ophthalmologist interviews were conducted via videoconference and two via telephone. Participants were generally located in their workplaces but no other people were present during the interviews. In line with the ethical clearance obtained, all participants were emailed copies of the consent form beforehand, and their consent was audio-recorded before starting the interviews. All interviews were audio-recorded and the researcher also took notes during each interview. The average duration of the interviews was 36 minutes. A semistructured topic guide (Supplemental Appendix 1) was used for all interviews; the design was informed by NPT.^24,26,32^ Five interviews were transcribed verbatim by the study researcher to aid familiarisation and immersion. The remaining interviews (n = 19) were transcribed using Scrintal Software and were checked for accuracy and anonymized by the researcher.

### Data analysis

Data analysis was informed by NPT.^24,26,32^ The recently published NPT coding book was used to structure the data analysis.^27^ The coding framework consists of three main domains (contexts, mechanisms, and outcomes) that include 12 primary constructs (strategic intentions, adaptive execution, negotiating capacity, reframing organisational logics, coherence building, cognitive participation, collective action, reflexive monitoring, intervention performance, relational restructuring, normative restructuring and sustainment). The coding framework also includes 16 subconstructs related to constructs in the mechanism domain. SA began data analysis by reading the interview transcripts several times to become familiar with the full dataset. Data was then coded deductively but broadly to the four primary constructs that informed the interview topic guide (coherence, cognitive participation, collective action and reflective monitoring), supported by nVivo 12 qualitative data analysis software. These constructs align with the four primary constructs that fall under the *mechanisms* domain in the NPT coding book. Transcripts were re-read to explore the relevance of the data to the *contexts* and *outcomes* domains, as the interviews had not specifically focused on these domains. Four primary constructs were found relevant, and data were coded to these constructs: strategic intention and adaptive execution from the *contexts* domain, and intervention performance and sustainment from the *outcomes* domain. Transcripts were then re-read and coded in more depth to subconstructs, where data supported that level of detail. For example, in coherence, data were coded to the internalisation and differentiation sub-constructs. Primary and secondary constructs identified in this study are summarised in Table 1. Data that did not fit the framework were coded separately and grouped under a theme called miscellaneous. This data was not included in the final analysis as it was not relevant to the study questions. The coding strategy was led and implemented by SA and discussed regularly with AB. Three themes (contexts, mechanisms and outcomes) and eight sub-themes (strategic intention, adaptive execution, coherence, cognitive participation, collective action, reflective monitoring, intervention performance and sustainment) are presented in the results section.

**Table 1.** Primary and secondary normalisation process theory (NPT) constructs identified in this study.^a^

Domain	Primary constructs	Secondary constructs
Contexts: Events in systems unfolding over time within and between settings in which implementation work is done	Strategic intention: How do contexts shape the formulation and planning of interventions and their components?	
	Adaptive execution: How do contexts affect the ways in which users can find and enact workarounds that make an intervention and its components a workable proposition in practice?	
Mechanisms: Motivate and shape the work that people do when they participate in implementation processes	Coherence: Participants contribute to enacting intervention components by working to make sense of its possibilities within their field of agency	Differentiation: How do people distinguish interventions and their components from their current ways of working?
		Internalisation: How do people construct potential value of interventions and their components for their work?
	Cognitive participation: Participants contribute to enacting intervention components through work that establishes its legitimacy and that enrols themselves and others into an implementation process	Enrolment: How do people join in with interventions and their components?
		Activation: How do people continue to support interventions and their components?
	Collective action: Participants mobilise skills and resources and make a complex intervention workable	Interaction workability: How do people do the work required by interventions and their components?
		Relational integration: How does using interventions and their components affect the confidence that people have in each other?
	Reflexive monitoring: Participants contribute to enacting intervention components through work that assembles and appraises information about their effects and utilise that knowledge to reconfigure social relations and action	Individual appraisal: How do people individually assess interventions and their components as worthwhile?
Outcomes: The practical effects of implementation mechanisms at work	Intervention performance: What practices have changed as the result of interventions and their components being operationalised, enacted, reproduced, over time and across settings?	
	Sustainment: How interventions and their components can be incorporated in practice?	

a Descriptions provided as per the NPT codebook by May et al.^26^

## Results

We structure our findings according to the top two levels of the constructs summarised in Table 1.

### Context

#### Strategic intentions

Most participants raised problems in the referral processes and suggested various approaches to overcome these issues.

One of the referral problems mentioned by many optometrists is the GP referral pathway. Many participants believed that sending the referrals to GPs instead of directly sending them to secondary care is inefficient in terms of time and is a significant hindrance. They felt that GPs lack the necessary knowledge or the right equipment to inform the referral decision to secondary care. Inefficiencies in the processing of referrals were also reported by many participants.

Cutting out the so-called ‘middleman’ and directly referring patients to secondary care was suggested by many participants to improve the efficiency of the referral process.

*I’ll be asking the patient what's happened, so did you get the, they’re like, oh, I never heard anything. And then when we look into the bottom of it, it hasn’t been actioned at the GP, either the letter has been, and so many times patients have taken the letters and they had to come back several times saying, oh, it's been lost at the surgery, in my opinion, avoiding that route and going directly to a hospital or a triage services is probably would be better. (Optometrist 10)*

Optometrists also suggested providing more direct pathways to refer suspected retinal conditions as a potential way to improve referral efficiency. Currently, the only direct pathway that was commonly used by participants is the rapid access pathway to refer patients with suspected wet AMD. However, practices differed in how they referred patients, with some using emails or online forms while others used faxes. Some optometrists also mentioned that there is a lack of clarity on the pathways that should be used as trusts and clinical commissioning groups (CCGs) have different pathways and protocols, suggesting the need to standardise the referral pathways across optometry practices.

*Well, I think more of these pathways are useful. And then more, um, better links between the ophthalmologists and the optometrists in the community. Um, that would be helpful, but there are some more pathways, and they are trying. So but I think it’s so random between practices that it would be nice if it was standardised. You know, every little borough has their own little way of doing things. I work in two different places, you know, and I and I worked on [name of an area], I don’t anymore. But I was. And you know, all the pathways are so different wherever you go. It would be nice if there was some sort of standardisation because otherwise it’s a postcode lottery all the time. (Optometrist 9)*

Most ophthalmologists, on the other hand, believed that the quality of the referrals by optometrists needs to improve. Two of the main issues mentioned were having insufficient details on referrals and unclarity of the attached OCT images, which made it difficult for ophthalmologists to triage referrals appropriately. Referring patients unnecessarily to secondary care was another important issue mentioned by ophthalmologists, with one of the ophthalmologists suggesting that around 60% of the referred wet AMD cases are false referrals. Some ophthalmologists identified the limited knowledge and experience of some optometrists as a potential reason for this, identifying the need to train optometrists to improve the quality of the referrals.

*There is an issue which is a knowledge issue which is we get referred, uh, patients that do not need to be seen in the hospital setting. And this is a problem that we have tried to address, with sort of teaching sessions to the community, and we had a successful one, um, last year. (Ophthalmologist 5)*

Many optometrists recognised the need to improve their decision-making in handling referral cases; however, they identified issues that act as barriers. One major issue that was identified by most optometrists was not receiving feedback from secondary care regarding the referred cases. It was described as a ‘one-way communication system’ and a ‘pain of our lives’, and made some optometrists feel unvalued in the system. Two types of feedback were identified in the interviews that could potentially help optometrists improve their overall practice and care for patients. The first is feedback from ophthalmologists. Most optometrists identified the need to know more about the ophthalmologists’ assessment of their referral to help them improve future referral decisions and avoid sending patients to hospital unnecessarily. The second is regarding the status of the referral, that is, whether the referral was received or not.

*But in 35 years, I could probably count on one hand the number of letters I’ve got back from the consultant. And that, I think, has to change. (Optometrist 16)*

*I’m not going to hear back from that. So were those angles narrow? Did I do the right thing? Or was that, uh, was that a false positive referral that I did, um, you know, how would I know, because then the next time I’m in that situation, I can improve that. And for me, it's all about improving it for the future. (Optometrist 5)*

The limited knowledge or experience of some optometrists, particularly junior ones, is another factor that could affect the quality of the referrals.

Some optometrists reported that optometrists with less experience sometimes prefer to refer any suspected case to secondary care, as they were concerned about their registration. The wide availability of OCT machines in optometry practices also means that a large number of OCT images are produced. Although many optometrists and ophthalmologists recognised the importance of OCT in eye examinations, a few optometrists believed this could result in overburdening the hospital, particularly when some optometrists have limited knowledge in interpreting OCT results.

*So I imagine that the ophthalmologists that are getting an awful lot of what they call it victims of multi-imaging technology have you come across it, you know, basically, these people have been getting false positive referrals. They’re getting a false referral because the clinician is covering their back. Yeah, basically the same. I don’t know what that picture says, therefore, I’m going to send it. (Optometrist 14)*

Overall, it was evident from the interviews that participants are open to new approaches and solutions to improve the efficiency of the referral process and the quality of their referrals.

#### Adaptive execution

Optometrists and ophthalmologists reported several solutions that are currently implemented to overcome issues with the referral process.

Having a direct link with ophthalmologists via email or by observing them in clinic was identified by many optometrists in this study as one effective way to improve the quality of the referrals. This is because optometrists can use this pathway to consult with the ophthalmologists regarding the urgency of the referral and receive direct feedback regarding the case. Some optometrists also have access to peer support groups which they use to get input regarding suspected cases. In their view, this has the advantage of providing them with a quick way to get feedback regarding cases they are doubtful about, particularly in the absence of links with clinicians in hospital. Most ophthalmologists also recognised the importance of having direct links with optometrists, with some taking the initiative to write back to optometrists regarding the clinical findings as they considered it an opportunity for educating optometrists. However, one of the barriers that ophthalmologists face is the dynamic nature of the optometrist community, with continuous locum movement and newly graduated optometrists joining. This is the same barrier encountered when they started training programmes to educate community optometrists on pragmatic ways to interpret OCT images.

*But by giving the feedback to them to state, these are the reasons why I think this is not urgent would be a learning process. And, uh, it has worked in some ways, but because there is quite a bit of a locum bank of opticians who come through, it has been an issue. (Ophthalmologist 6)*

Another solution that has been implemented in several practices is using online systems to refer patients to the GP instead of the paper referral. According to some optometrists, these systems help improve the efficiency of the referral process as it allows them to upload referral letters and attach images. This way they are reassured that their referrals ‘are not lost in the system’. However, a few optometrists reported not using these online systems due to not getting feedback about their referred case, and because sometimes GPs are not registered on these systems.

*Not all GPs are registered on the platform yet. So we basically type in the name, if their name comes up, then we can send it via [the name of the online referral system], if it’s not on there we’d still have to do the traditional method of writing it and sending it with the GP or posting it to the GP ourselves. (Optometrist 5)*

Some practices also had access to central referral hubs. These hubs act as a single point of access to secondary care. It involves hospital-level optometrists or ophthalmologists triaging the referral and deciding on its urgency. It might also involve repeating some of the tests like OCT before the patient is referred to hospital. Generally, optometrists who have sent referrals through these hubs thought it was a positive step and could improve the accuracy of referrals. These systems bypass the GP, although it notifies them, which is another advantage of these systems from optometrists’ point of view.

Overall, there were several ongoing initiatives to improve the efficiency and quality of referrals. Optometrists and ophthalmologists who were involved in the implementation of these solutions were generally positive about them. However, except for the aforementioned rapid access pathway for suspected wet AMD cases, there was not a large-scale solution that all practices implemented or had access to.

### Mechanisms

#### Coherence

Some common characteristics were mentioned by optometrists, particularly those in the control group, which they expect to see in a teleophthalmology technology to improve the overall referral process. One of these characteristics is the ease of use and time efficiency of the platform where they can upload the OCT images and the referral letter or referral information into the system, ideally on the same screen. The information on the referral is also expected to be streamlined to the currently used referral system in the United Kingdom, as any additional step could be considered an added burden to their routine. Additionally, the system should allow them to have direct communication with ophthalmologists; however, optometrists’ expectation on the means of communication varied based on their prior exposure to teleophthalmology platforms. Those with previous experience expected the communication to be online or via email, whereas those with limited experience believed it would involve telephoning ophthalmologists as the term teleophthalmology implies. Optometrists expected that having a direct link with ophthalmologists in a teleophthalmology platform would provide them with the opportunity to consult with them regarding the referral decisions. They also expected to receive feedback via the teleophthalmology platform, particularly about the ophthalmologist's clinical decision, as would be included in a discharge letter.

*Yeah, yeah, maybe it’s just an ideal thing would be a good and easily accessible user-friendly system or platform that is time effective that where the optometrist can upload or communicate with ophthalmologists or other experts: nurses, etcetera and get maybe sometimes feedback, ideally within a few days if there’s uncertainty or queries or if, you know, making referrals to the right place in a good time. (Optometrist 18)*

Several perceived benefits of teleophthalmology were identified by optometrists. Many believed that receiving immediate feedback from ophthalmologists via teleophthalmology would reduce unnecessary visits to hospitals as it would help them improve their practices and avoid mistakes. They also believed that they would be valued and trusted more by ophthalmologists, especially when they diagnose correctly. Several optometrists believed that implementing teleophthalmology would improve patients’ experience of the referral as it would prevent them from going to hospital unnecessarily, alongside keeping them informed about their care. Saving on NHS cost by bypassing the GP and reducing patients’ unnecessary visits was thought to be another potential benefit by some optometrists.

*Well, it’s just a case of, knowing what you’re doing, where we’re going, of course, the finesse is coming to it, and to reassure patients that they are being seen by ophthalmologist, and to give us credit that you know we have diagnosed correctly and we are referring in a valid and timely manner. Um, as I say, sometimes we do waste GPs’ time by doing the referral to them, we are sending the referral to them when we could do it directly and more efficiently, so to waste and stop wasting national health service time. (Optometrist 1)*

Some barriers were identified by optometrists that they thought they may face in the implementation of any teleophthalmology technology. Many optometrists believed that poor IT infrastructure such as the lack of hardware or required software to operate new teleophthalmology platforms that some community optometry practices have might be a barrier. Also, the lack of interoperability between the different systems used in practices, particularly those used to manage the OCT machines and the patient details, could pose challenges for implementing teleophthalmology platforms. Some optometrists also thought that the IT skills of optometrists, especially ones less familiar with new technologies, might be a potential barrier. The cost of the OCT machine and not receiving the appropriate remuneration were perceived by some optometrists as potential barriers to implementing teleophthalmology, particularly in less well-off areas.

#### Cognitive participation

Most optometrists were ready to engage with the HERMES teleophthalmology platform as they were familiar with teleophthalmology platforms or online referral systems, and it also addressed their desire to overcome referral issues. All optometrists in the intervention arm had received training on using the HERMES teleophthalmology platform for the study, supplemented with flowcharts that explained the steps they needed to follow when referring a patient via the platform. However, in practice, several optometrists faced difficulties in following the process described in the flowchart, particularly for the first few patients. The main issues raised by optometrists regarding the process of referral were specific to conducting research in practice. For example, many optometrists found that explaining the study and consenting patients was ‘tedious’, ‘the longest part’, or ‘long-winded’. Anonymizing the OCT scan was another issue that many optometrists found laborious. However, some optometrists recognised that these steps are specific to the research context and not included in real practice. In addition, many optometrists found filling the online referral form (extended from that used in routine referrals) to be time-consuming, with some estimating the time to be around 30 minutes. Other issues raised by many optometrists were that the referral form (or questionnaire) included repetitive questions, and that they were also asked to answer every question although some were irrelevant to the referred case.

*But I think the whole process from the questionnaire to the, um, take the images twice and then sending them across, you know, that is cumbersome. It’s a laborious process. (Optometrist 11)*

The support provided by the project manager was essential to those who faced any difficulty in the process, particularly those with limited IT skills. Sharing feedback with the project manager resulted in some changes to the process, such as telephone consenting patients and pre-filling the consent form.

#### Collective action

Optometrists employed different strategies to incorporate doing the referral on the platform. One of the main changes to their practice was adding more time to their regular eye examination appointments, particularly for those patients who they believed might need a retinal referral. Many optometrists completed the referral when the patient was not present, taking advantage of their ability to access the platform remotely. This included completing the referrals before or after clinics, during their lunch breaks or on days off. Another change that was implemented in practice was dividing the eye examination and the referral process. For example, a few optometrists mentioned that when identifying a referrable case to HERMES, they would ask the patient to do the OCT test and consent on another visit. It was also an opportunity to provide patients with more time to think about the study. Other optometrists prioritised carrying out an OCT scan for their patients when they were time-constrained, particularly if they believed the referral was urgent. Some optometrists depended on their colleagues to help them with some of the steps such as anonymisation of the patients’ details. Those with less experience in referring patients focused on consenting patients and completing the online form; however, the final referral decision was jointly made with a senior member of staff.

*it is just time I think, I added time, I have a folder for all the consent forms and the info sheets and everything. I’ve had to work from home and log in and just to complete it because, you know, some of these referrals need to be done soon, quickly you know we can’t wait for some time. You know two or three working days later, so it’s going to be done quickly. So had to work overtime in that sense to sort of action it and get it done. (Optometrist 6)*

Ophthalmologists received email notifications when a referral had been uploaded on the platform, alongside an email from the study coordinator. Most ophthalmologists actioned the referral as soon as they could access the platform and review the referral. This depended on their clinic timings and the time of the day they received the referral. Like optometrists, ophthalmologists were able to access the platform remotely and review some of the referrals. However, some OCT images could only be viewed in a clinical setting, which according to one ophthalmologist was ‘quite frustrating’. Additionally, there was some variations in how referrals were managed in different sites. In some cases, the referrals were triaged based on whoever saw them first, while at other sites a specific person was assigned to review all referrals, with another person covering for them during their absence.

#### Reflective monitoring

There were some common factors that optometrists used to judge the value of the teleophthalmology platform. Receiving feedback from secondary care regarding their referred case was one factor that several optometrists valued. However, they reported inconsistencies regarding feedback received via this pathway. Some optometrists received regular feedback regarding the cases referred through the platform. They were generally satisfied about it, given that in usual practice they rarely receive feedback about referred cases. It also provided them with reassurance that what they had referred for was correct. However, in some cases, optometrists would have liked more detailed feedback. Other optometrists reported not receiving feedback on any of their referred cases. In addition to the importance of feedback for their professional development, optometrists required this feedback to inform patients regarding the status of their referral.

*That would be a critical factor for us that there was, uh, very clear feedback both to us and the patient. I’ve not really had confirmation of any of the sort of provisional diagnosis that we’ve made on the HERMES, whether we refer them correctly or not. And that would be very useful for us. That’s very useful for learning. Because generally if you say to someone you have a problem, it would worry them, even if it’s not serious, it still concerns people, so telling the patients that they will be contacted in a couple of days give them this reassurance. (Optometrist 7)*

The speed of sending a referral, and having assurance that a referral decision would be made within 48 hours through the teleophthalmology platform, was considered an advantage by many optometrists. Many encouraged patients to send their details through the HERMES platform based on the speed of this referral route compared to the traditional pathway. Therefore, some optometrists felt disappointed when the process took longer than expected, which led them to question the value of this referral pathway. Additionally, a few believed that this advantage is because of the study environment, and this might not be case when teleophthalmology is implemented in practice, as the volume of referrals will be larger.

Ophthalmologists, in general, had positive experiences triaging referrals via the platform. Most ophthalmologists found the platform straightforward and easy to use. Time was an important factor that ophthalmologists used to assess their experience triaging referrals on the platform, and the majority reported the time taken to use the platform was reasonable. Although a few ophthalmologists believed it took longer than usual practice because of the need to review OCT images, this was considered acceptable as it helped them achieve high-quality decisions. Despite their overall positive experience, ophthalmologists suggested some improvements for the platform. One suggestion was adding a question regarding the duration and details of symptoms, which would help them decide on the urgency of the referral. Another suggestion was to only receive a notification to triage complete referrals, as they are currently getting a notification for each step performed by the optometrist. Improving the way a specific OCT machine interfaces with the platform was another point mentioned, which is a matter of user-interface design optimisation. However, suggestions to improve the platform were viewed as minor, while addressing the barriers that optometrists may face when using the platform was considered more significant by some ophthalmologists.

### Outcome

#### Intervention performance

The views on the intervention performance came largely from sites that had referred several patients to the HERMES study. Ophthalmologists’ views were mixed regarding the quality of the referred cases using the teleophthalmology platform as compared to the traditional pathway. Some ophthalmologists believed that their decisions using the teleophthalmology platform were better because they had the required information to triage effectively, and that the quality of the referrals received via the platform improved compared to what they usually receive from community optometrists. This is because the referrals received via the platform had the optometrists’ provisional diagnosis, whereas in usual practice optometrists are not required to propose a diagnosis. However, a few ophthalmologists thought this might be because of the study environment and that optometrists felt their decisions would be scrutinised. A few ophthalmologists shared that some information in the referrals were missing and that optometrists were still sending cases that should not be referred.

*they are more cautious of referring let's say silly things, for example, and unexplained, visual loss without any scan, uh, without the pathology and OCT scan or at a photograph. So they don't refer it, of course, to the medical retina. Uh, but still, you refer, you have cases that they have just a single, uh, epiretinal membrane or very mild lamela hole that cannot progress very quickly and possibly doesn't need to be seen at the secondary care environment unless it shows progression through the time. (Ophthalmologist 4)*

Optometrists’ views on the impact of using the teleophthalmology platform on their usual practice were also mixed. Some optometrists believed that using this pathway improved the efficiency of the referral process, given the speed of the review process. A few optometrists believed that using this pathway increased confidence in their referral decisions, and alleviated some of the pressure they experienced, particularly when those referrals were accepted. Conversely, several optometrists did not think that using the teleophthalmology pathway had an impact on what they would normally refer. They also felt that the main change in their practice was adding time to their normal routine. In terms of relationship with secondary care, some optometrists believed that using this pathway had consolidated existing relationships with ophthalmologists as they had regular communication and updates regarding the referred cases. However, other optometrists did not believe it had an impact on their relationships with ophthalmologists, due to existing good relationships or limited contact with secondary care.

*I mean, it's a referral, ok, it makes no difference, ok, I see a problem, I discuss it with the patient and say listen I'm not happy with it, I think we need to see a specialist about it, it’s, when I realized that something has to be done, it's not oh no what I do, it's just knuckle down and do it. (Optometrist 3)*

*besides learning to anonymize the scans and, you know, setting up the obvious platforms on the computer, I don't think we've done anything different. (Optometrist 9)*

#### Sustainment

Some factors were identified that could contribute to the sustainability or the incorporation of the platform in practice. Some of these factors were related to the platform such as ease of use, the time to complete the online referral and offering direct communication between optometrists and ophthalmologists. These factors were like those optometrists expected in any teleophthalmology platform implemented in practice. Some ophthalmologists believed that the platform should be incorporated into patients’ electronic records. The platform should also allow them to send feedback to patients, in addition to optometrists. Some factors were related to the optometrist's skills. For example, some optometrists and ophthalmologists believed that providing optometrists with hands-on training on how to interpret OCT images would improve the quality of their referrals via the platform. The limited IT skills of optometrists, particularly ones less familiar with new technologies, was often mentioned as a potential barrier and should be addressed when implementing the platform.

*so I think the barriers for me have just been that although I'm fairly articulate with the some of the bits of computerization, I'm not as well practiced as what the younger people are, so for the younger practitioners I think they'll tag to it in no time at all, so and it'll just ensure that everybody is able to get referral in a much quicker, more economical, a more advanced format. (Optometrist 3)*

Other factors were largely related to finances. Providing remuneration to optometry practice to purchase OCT machines was considered an important factor to facilitate the adoption of teleophthalmology platforms in practice. Persuading CCGs and NHS Trusts to invest in community optometrists and new direct pathways as well as contracting out services in the community were suggested as other factors that could aid the integration of teleophthalmology pathways in practice. Increasing patients’ awareness of the importance of doing an OCT in their eye examination was also considered essential to implement the teleophthalmology pathway in practice.

*Very difficult for me. Uh, first of all, you have to persuade all managers, okay. Not only the managers, but also the CCGs to, uh, the CCGs to have this platform on. The second thing is, uh, this is one point from the secondary care, so we were getting involved in each county to go and persuade them to pass through, into the national system as we have passed the test. But on the other side now of the optometrists, I mentioned, uh, also some problems that we face, and there are big changes of optometrists like [name of high street optometry practices] that they might not accept this and also, uh, the representatives of them in the CCGs. Yeah, So it's not because the clinicians don't like or optometrists don't like. Sometimes it's a big it's a big game behind that. (Ophthalmologist 4)*

## Discussion

Using NPT as a theoretical approach, our study has highlighted several factors that could influence the adoption of teleophthalmology platforms in the referral process by optometrists and ophthalmologists or, alternatively, lead to the abandonment of these platforms.

Most optometrists recognised the value of using the teleophthalmology platform and were generally ready to engage with it. For example, they identified reducing the number of referrals to secondary eye care and improving patients’ experience of the referral as potential benefits of using the platform, consistent with existing literature.^20,21^ Understanding the value of the new technology and its potential benefits is often an important facilitator in accepting new technologies.^33,34^ Similar studies show health professionals’ openness to new technologies that improve workflows and patient care.^35,36^ However, shortcomings in meeting support expectations can serve as a barrier.^35^ Optometrists in our study expected teleophthalmology platforms to facilitate communication with ophthalmologists, providing an opportunity to discuss referred cases and enhance the quality of their future referrals. However, feedback sometimes fell short of their expectations, leading many to feel that the platform did not improve their referral cases. Ophthalmologists, on the other hand, generally had a more positive experience when using the teleophthalmology platform, noting improved decisions due to having the necessary information to triage effectively. These findings suggest that the teleophthalmology platform may have prioritised ophthalmologists’ perspective to address over-referral issues, potentially overlooking optometrists’ viewpoints. They also underscore the importance of identifying key stakeholders and involving them during the early stages of technology design and development, before implementation.^13,29,37^ This can help in identifying user expectations and designing technology that meets them, enhancing its potential for future adoption.

Another important factor affecting the experiences of health professionals is the time it adds to their current work routine. In our study, this may have contributed to the relatively positive experience that ophthalmologists had when interacting with the platform, with the majority finding the additional time reasonable. In contrast, many optometrists found filling out the form to be time-consuming, leading to a less positive experience with the platform. Time constraints and the impact on health professionals’ workload are important considerations when developing a teleophthalmology platform.^34,36,38^ For ophthalmologists, the additional time was acceptable because it improved their decision-making process. This suggests that health professionals might be willing to incorporate some extra time into their usual practices if it leads to an overall improvement in patient care rather than merely adding administrative burden.

Individual factors played a critical role in either facilitating or inhibiting the adoption of the teleophthalmology platform. Among optometrists, there were differences in how they adjusted their work routines, with some prioritising conducting the OCT scan and others dividing the eye examination differently. Additionally, some optometrists, especially those with limited IT skills, required extra support to complete the online referral form. The success of technology implementation may depend on how well these differences are considered when designing, developing and implementing the technology. In our study, the HERMES trial manager played a crucial role during the implementation stage by providing continuous support to optometrists. Providing training and support to potential users of the digital health technology are widely recognised as being essential for successful implementation.^33,34,38,39^ This can help health professionals overcome barriers related to unfamiliarity with the technology and equip them with the necessary skills for its use.^39^ Offering training and support can also help health professionals in integrating technology into their work routines.^33^ However, one might question the feasibility of providing the level of intensive support observed in our study in a non-trial setting, particularly considering that many digital health interventions are implemented in resource-constrained settings. One approach to address this issue is to identify individuals who require support and assess the extent of support needed, taking into account the technology complexity and available resources. As demonstrated in our study and in other research,^38^ health professionals have varying support needs and not everyone requires intensive support or training.

Our study emphasised the importance of understanding the implementation context to identify facilitators and barriers to adoption. For example, the issue of referrals is well-recognised, and both optometry practices and secondary care were keen to address them by implementing various solutions. However, most solutions were at a small-scale, and except for the rapid access pathway to refer wet AMD cases, there was no large-scale digital health intervention or referral platform that all practices implemented or had access to. This highlights the complexity associated with implementing digital health interventions and that factors unrelated to individuals’ motivation to solve the problem or readiness to use the technology must be considered for successful large-scale implementations of such interventions. Participants in our study mentioned key factors that may act as barriers to integrating the teleophthalmology platform into their usual practice, including the cost of the OCT machine, poor IT infrastructure, lack of interoperability between OCT machines and optometry practice systems, and the need for investment in community optometry services. Some of these factors are already identified in the literature as potential barriers to implementing healthcare technologies.^3,36,39,40^ Additionally, implementing the platform alone might not result in the desired improvement in the referral quality. Initiatives like training optometrists on interpreting OCT images may be necessary to achieve a significant improvement in the quality of the referral. This finding aligns with research conducted by Ramchandran et al.,^36^ who identified the importance of hands-on training in eye health to facilitate engagement with teleophthalmology in diabetic retinopathy surveillance. To achieve large scale implementation of the teleophthalmology platform, addressing these organisational factors is essential. Otherwise, there is a risk of limiting its implementation to a small scale or even potential abandonment of the technology, which is a common risk associated with digital health interventions.^3,7,8,41^

Another important consideration is the potential impact of research processes on participants’ engagement with digital health technology. In our study, some optometrists found research processes such as consenting patients to be time-consuming. This may have acted as a barrier to their engagement with the research and the digital health intervention. Previous research supports this observation,^41^ indicating that research processes can lead potential participants to decline involvement in interventions. Given the equal importance of involving end users and complying with these research processes, it is important to find ways that strike a balance between the two. One potential solution identified in our study is obtaining participants’ consent for the HERMES trial via telephone which seemed to be well-received by optometrists.

**Figure 2.**
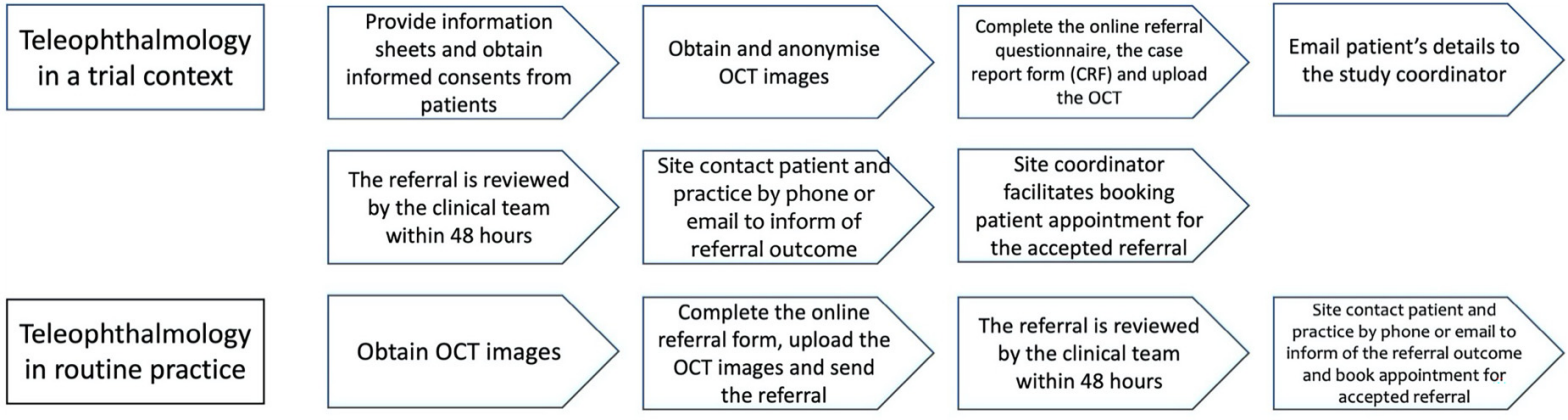
Teleophthalmology pathway in a trial setting versus routine practice.

Additionally, our findings, consistent with Perski and Short,^41^ highlight the importance of differentiating between barriers that stem from the technology itself and those that arise from the research process. For instance, optometrists’ experiences with the teleophthalmology platform might have been more positive if the research processes had been time-efficient and aligned with their needs. As described above, the HERMES teleophthalmology platform was implemented in the context of a trial, with additional trial-specific steps. Optometrists’ experiences may be more positive in real-life implementation since these trial-specific steps will not be necessary. Figure 2 illustrates the difference between the teleophthalmology pathway in a trial setting and the expected pathway if integrated into routine care, highlighting the steps that would be omitted.

## Reflections on NPT as a theoretical framework for this study

One of the benefits of using NPT as a theoretical approach is that it not only identifies the barriers but also helps in understanding why these barriers develop and where they exist in the acceptability process of the technology. For instance, in our study, one of the main barriers to optometrists accepting the referral platform could be attributed to the mismatch between their expectation of what the technology should do and the actual functionality of the technology, such as the feedback component of the referral platform. Similarly, most of the barriers identified by optometrists were related to practical aspects of using the technologies such as the added time to complete the online referral form rather than their readiness to engage with it. As demonstrated in our study, most participating optometrists were motivated to overcome referral issues and engage with the technology.

Using the NPT code book^27^ facilitated structuring the findings and our study serves as an exemplar of utilising this codebook. However, it is important to note that the interviews were conducted before the codebook was published. Questions were primarily structured around the four core constructs of NPT (coherence, cognitive participation, collective action and reflective monitoring). Our understanding of NPT and the development of the topic guides were influenced by key references on NPT^24,26,32^ identifying these as the theory's main constructs. Despite addressing questions related to the implementation context and potential impacts on participants’ practices during interviews, questions were not initially framed in alignment with the NPT codebook. We cannot retrospectively assess how different our findings would have been if the questions had been framed in terms of the codebook.

## Strengths and limitations

As well as the general observations about the evolution of NPT and its reification in the codebook, it is also noteworthy that the ‘sustainment’ subconstruct in NPT typically focuses on factors that contribute to ‘normalising’ or incorporation of the intervention into practice, rather than the expected factors.^27^ However, given the short time the platform had been implemented at the time of the interviews, most of the identified factors were related to the perceptions of optometrists and ophthalmologists regarding what would sustain the intervention, rather than what was actually contributing to its sustainment.

Because the NPT coding book was used to structure the data analysis, we did not develop concepts directly from the data, as we did in our complementary inductive analysis.^31^ In practice, we found almost no new concepts or insights in the final few transcripts, and nothing that challenged the analysis up to that point, so we are confident that the analysis is consistent with the whole dataset.

A strength of this study is including the lived experiences of both optometrists and ophthalmologists of the platform. This enabled us to compare and contrast their experiences, revealing differences and identifying barriers faced by either or both groups. However, it is noteworthy that some of the participants had prior experiences with teleophthalmology platforms which could have influenced their experience with the platform under investigation.

## Conclusion

In this study, we emphasised the need to consider multiple factors when developing and implementing teleophthalmology platforms, especially if we want to see the technology adopted and normalised at a large scale and address the issue of over-referrals to secondary eye care. These factors relate to the technology, such as the time it takes to complete the referral on the platform, to the individuals such as their levels of IT skills, and organisational considerations like cost of equipment, IT infrastructure and training and investing in community optometry practices. Additionally, we emphasised the importance of involving key stakeholders in the early stages of technology design and development, as well as during implementation to ensure that the developed technology matches their expectations of support. The importance of designing and implementing research processes that are time-efficient can also be considered as key for active and successful engagement of health professionals with the digital health intervention. Overall, NPT proved to be a useful approach to untangle some of the complexities associated with the implementation of digital health interventions, using the HERMES teleophthalmology platform as an example.

## Supplemental Material

sj-docx-1-dhj-10.1177_20552076241303812 - Supplemental material for Implementing a teleophthalmology referral platform in routine practice: Understanding a digital health intervention implementation using normalisation process theory Supplemental material, sj-docx-1-dhj-10.1177_20552076241303812 for Implementing a teleophthalmology referral platform in routine practice: Understanding a digital health intervention implementation using normalisation process theory by Sarah Abdi, Dilisha Patel, Josie Carmichael, Konstantinos Balaskas and Ann Blandford in DIGITAL HEALTH

sj-docx-2-dhj-10.1177_20552076241303812 - Supplemental material for Implementing a teleophthalmology referral platform in routine practice: Understanding a digital health intervention implementation using normalisation process theory Supplemental material, sj-docx-2-dhj-10.1177_20552076241303812 for Implementing a teleophthalmology referral platform in routine practice: Understanding a digital health intervention implementation using normalisation process theory by Sarah Abdi, Dilisha Patel, Josie Carmichael, Konstantinos Balaskas and Ann Blandford in DIGITAL HEALTH
