# Supplementary material for: Implementing a teleophthalmology referral platform in routine practice: Understanding a digital health intervention implementation using normalisation process theory
Source: Digit Health. 2025 Jan 31;11:20552076241303812. doi: 10.1177/20552076241303812 (PMC11786278; doi:10.1177/20552076241303812)
Supplement: sj-docx-1-dhj-10.1177_20552076241303812 - Supplemental material for Implementing a teleophthalmology referral platform in routine practice: Understanding a digital health intervention implementation using normalisation process theory [file sj-docx-1-dhj-10.1177_20552076241303812.docx]

HCI study

Interview topic guide

# Information and consent

- - Briefly re-introduce the topic, the purpose of the overall project and the specific aim of the HCI study
  - Obtain informed consent (if not taken in advance) and permission to be audio/video recorded

# Introduction

Thank you for taking part in this interview. We really appreciate you taking the time to talk with us. As I explained earlier, we are keen to talk to you about the use of new teleophthalmology technologies in the referral process between community optometry practices and hospital eye services. We want to hear your views on these technologies, and what factors you think may influence their implementation in real life**.** There are no right or wrong answers, so feel free to tell us what you think, and if there is something that needs to be improved.

The interview is likely to take around 30-45 minutes. Your participation is voluntary, and you can withdraw from the study at any time.

# Background Information

- First of all: I will start by asking you a few questions to know more about you.
- What is your professional background?
- How long have you been working in community optometry practices/hospital eye services?
- How often do you refer patients with retinal conditions to hospital eye services? (in a week what is the average number of cases identified with retinal conditions)
- Can you explain to me the different referral pathways that you follow when you identify someone with a suspected retinal condition (usual not through the teleophthalmology platform)
- What do you think of the referral process?
  - What are the issues or barriers that you face when referring patients?
- How do you think the referral process can be improved?

# Teleophthalmology platform

## Coherence

- What do you think a teleophthalmology platform is? How different is it from the traditional system?
- What would you like to see in a teleophthalmology platform?
- How do you think a teleophthalmology platform would benefit/improve the referral process for eye conditions?
- What barriers do you think there will be to using a teleophthalmology platform in the context of referrals?
- What do you think would need to change to incorporate a teleophthalmology platform in your practice?

## Cognitive participation

- How long have you been using this new teleophthalmology platform?
- Can you describe to me the steps you follow when you refer a patient with a suspected retinal condition through the new teleophthalmology platform?
- How long does it take you to do a referral on this platform? What do you think of that?
- What did you like about this new platform? Why?
- What did you not like about this new platform? Why?
  - What were the issues that you faced when using the platform?
  - Do you have any concerns regarding the use of this platform?
- What type of support did you receive to use this platform (e.g., training, support staff)?

## Collective action

- What changes did you have to make in your work routine to incorporate this new technology?
- How will this new platform fit in your current practice?
- How do you think this new platform will impact/improve your practice, patient care and your working relationship with secondary care?

## Reflective monitoring

HCI study

- What do you think will be the benefits of this new teleophthalmology platform on the referral process, both short term and long term?
- How do you think this new teleophthalmology platform can help reduce unnecessary referrals?
- How do you think this new teleophthalmology platform can help improve the quality of referrals?
- What barriers/disadvantages do you think there will be to using this new platform on the referral process?
- What do you think would need to change for this technology to be successfully embedded in routine eye practice (e.g., training…)?

# End of the interview

We have reached the end of the interview. Would you like to share any more ideas or thoughts?

Thank you so much for taking the time to participate in the study, we will use this information to inform the design of future teleophthalmology services.
